# Optimization of psoriasis assessment system based on patch images

**DOI:** 10.1038/s41598-021-97211-9

**Published:** 2021-09-13

**Authors:** Cho-I. Moon, Jiwon Lee, HyunJong Yoo, YooSang Baek, Onseok Lee

**Affiliations:** 1 Department of Software Convergence, Graduate School, Soonchunhyang University, 22, Soonchunhyang-ro, Asan City, Chungnam-do 31538 Republic of Korea; 2 Department of Computer Science & Engineering, Graduate School, Soonchunhyang University, 22, Soonchunhyang-ro, Asan City, Chungnam-do 31538 Republic of Korea; 3 Department of Medical IT Engineering, College of Medical Sciences, Soonchunhyang University, 22, Soonchunhyang-ro, Asan City, Chungnam 31538 Republic of Korea; 4 Department of Dermatology, Guro Hospital, Korea University College of Medicine, Seoul, 08308 Republic of Korea

**Keywords:** Health care, Medical research

## Abstract

Psoriasis is a chronic inflammatory skin disease that occurs in various forms throughout the body and is associated with certain conditions such as heart disease, diabetes, and depression. The psoriasis area severity index (PASI) score, a tool used to evaluate the severity of psoriasis, is currently used in clinical trials and clinical research. The determination of severity is based on the subjective judgment of the clinician. Thus, the disease evaluation deviations are induced. Therefore, we propose optimal algorithms that can effectively segment the lesion area and classify the severity. In addition, a new dataset on psoriasis was built, including patch images of erythema and scaling. We performed psoriasis lesion segmentation and classified the disease severity. In addition, we evaluated the best-performing segmentation method and classifier and analyzed features that are highly related to the severity of psoriasis. In conclusion, we presented the optimal techniques for evaluating the severity of psoriasis. Our newly constructed dataset improved the generalization performance of psoriasis diagnosis and evaluation. It proposed an optimal system for specific evaluation indicators of the disease and a quantitative PASI scoring method. The proposed system can help to evaluate the severity of localized psoriasis more accurately.

## Introduction

Psoriasis is a chronic inflammatory skin disease characterized by papules and plaques covered with silver-white scales. It occurs regardless of age or sex. It has a worldwide prevalence of approximately 3%^1^. Although psoriasis mainly affects the skin, it is associated with psoriatic arthritis, heart disease, diabetes, and mental depression. As keratinocytes proliferate due to genetic factors and problems with the immune system, psoriasis presents with skin thickening, scaling, and erythematous changes.

Currently, the diagnosis and evaluation of psoriasis rely on ocular inspection and palpation by a dermatologist. The severity of psoriasis and changes before and after treatment are evaluated using the psoriasis area severity index (PASI) score, a representative psoriasis evaluation tool^2^. In some countries (e.g., the Republic of Korea), it is used as a reimbursement criterion for biologics in severe psoriasis and is adapted to the medical care of patients with severe psoriasis. The PASI score is evaluated based on four variables: erythema (redness), thickness, scaliness, and percentage of body surface area covered. The severity of psoriasis is scored as shown in Eq. (1) below by assigning a weight to the body area^3^. E, T, S, A mean erythema, thickness, scale, and area of psoriasis, respectively, and h, u, l, and t indicate the head, upper extremities, lower extremities, and trunk.

1 $$PASI \,\,score=0.1\times \left({E}_{h}, {T}_{h}, {S}_{h}\right)\times {A}_{h}+0.2\times \left({E}_{u}, {T}_{u}, {S}_{u}\right)\times {A}_{u}+0.3\times \left({E}_{t}, {T}_{t}, {S}_{t}\right)\times {A}_{t}+0.4\times \left({E}_{l}, {T}_{l}, {S}_{l}\right)\times {A}_{l}$$

However, because the PASI score relies on the subjective evaluation of the clinician, deviations in disease diagnosis may occur. Various computer-aided diagnosis (CADx) systems have been developed to quantify psoriasis disease evaluation^4–9^. These previous studies have dealt mainly with the segmentation of psoriasis areas and the classification of severity. Most of them conduct segmentation or classification studies independently^10,11^, thus targeting a single disease within the range of observation, or mainly dealing with the segmentation of disease areas with apparent differences in shape or color^12^. However, psoriasis has various forms depending on the severity of the disease. The boundaries are ambiguous, the characteristic difference is unclear, and several lesion areas are distributed within the observation range. Therefore, in practice, a system evaluating psoriasis must accurately detect lesion areas and simultaneously evaluate their severity. Therefore, we studied the segmentation and classification method that works robustly for various types of psoriatic diseases and attempted to prove the performance of the psoriasis evaluation system.

For a two-dimensional analysis of psoriasis, we focused on the characteristic changes of erythema and scale among the PASI score indicators according to severity based on the local region where the disease is distributed. We present a segmentation method and a severity classifier suitable for the diagnosis of psoriasis by performing a comparative evaluation of four segmentation methods and six multiple classifiers. First, for the segmentation of psoriatic disease areas, we used the interactive graph cuts (IGC)^13,14^ and level set method (LSM)^15,16^. These semi-automatic segmentation algorithms show high-level segmentation results in medical imaging, computer vision, and graphics. Simple linear iterative clustering (SLIC) superpixel-based segmentation^11,17^ and artificial intelligence model U-Net^10,18^ were used as an automatic segmentation algorithm. Through a comparative analysis of the segmentation performance of psoriasis, we propose a segmentation algorithm that operates robustly despite environmental noise or ambiguity of the disease. Next, to classify the severity of psoriasis, the machine learning method and six artificial intelligence models were compared and analyzed. These models were error-correcting output codes-support vector machines (ECOC-SVM), naïve Bayes (NB), k-nearest neighbor (k-NN), random forest (RF), adaboosting (AB), and deep neural network (DNN)^19,20^. The criteria to determine which method is best for classifying the severity of psoriasis are not clear and have not been established. Therefore, we present a classifier that performs best in classifying psoriasis severity by evaluating classifiers typically used in various problems with different strengths and weaknesses. In addition, as the severity of psoriatic disease increases, the development of erythema and scales is more evident. However, since erythema and scales coincide in the same disease, separating and evaluating these two features with the naked eye is difficult. Therefore, we constructed a new dataset by yielding patch images for erythema and scales based on the color characteristics of the diseased area. It is possible to develop a severity classifier that is more robust to the features of erythema and scale by stuyding patch images of erythema and scales of psoriasis.

In this study, a new dataset including patch images of erythema and scales was presented to improve the generalization performance of psoriasis diagnosis and evaluation and improve the severity classification performance for specific evaluation indicators of disease. In addition, we propose optimal disease segmentation and classification methods for a new psoriatic disease dataset.

## Results

**Figure 1:**
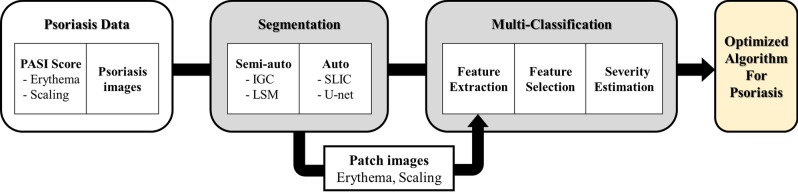
Outline of our proposed psoriasis severity assessment system. It shows the overall structure of the psoriasis severity assessment system proposed in this study. First, we compared the psoriasis segmentation results of the four segmentation methods and selected the segmentation methods with the best performance. We then obtained patch images of erythema and scaling using the best segmentation images and generated a new dataset including patch images. Next, we classified psoriasis severity based on psoriasis features (texture, color, and spectrum) using a new dataset. Through two stages, we present a segmentation method and a classifier optimized for psoriasis images.

In this study, the architecture of the proposed psoriasis severity assessment system consisted of two steps (Fig. 1). The first was the segmentation process of the psoriasis region. The accurate detection of psoriasis is an essential step in disease analysis. We evaluated and compared the performance of the four segmentation algorithms. Second, we classified the five severity levels of psoriasis (healthy, mild, moderate, severe, and very severe). Based on the best segmentation method results, we generated patch images of erythema and scaling features and constructed new datasets for psoriatic diseases. We trained six multi-label classifiers using the new datasets and performed a comparative evaluation to present the classifier with the best performance. Consequently, we propose the best segmentation algorithm and severity classifier for psoriasis that can be applied to the psoriasis diagnosis and evaluation system.

### Image segmentation for psoriasis

Detecting diseases within the observation range is an essential step in increasing the precision of diagnosis. Therefore, accurate segmentation of the psoriasis region is required. This segmentation process is a necessary step in the objective quantization of diseases. However, when the severity of psoriasis is weak or too severe and when the range of psoriasis lesions is expanding or when traces of the psoriasis region remain during treatment, the boundaries between the disease area and the surrounding normal area of the skin become ambiguous, thus making it difficult to detect the psoriasis region accurately.

We evaluated the performance of the segmentation methods for psoriasis was from the traditional techniques to state-of-the-art techniques. We used 70 images of psoriasis, which varied in shape and size according to severity, had multiple diseases within the observation range, and had various noises such as body curvature and shading. In our experiment, we used Automatic (SLIC superpixels and the U-Net model) and semi-automatic (LSM and IGC) segmentation algorithms. We used five similarity indicators to evaluate the segmentation performance of the four segmentation algorithms.

**Figure 2:**
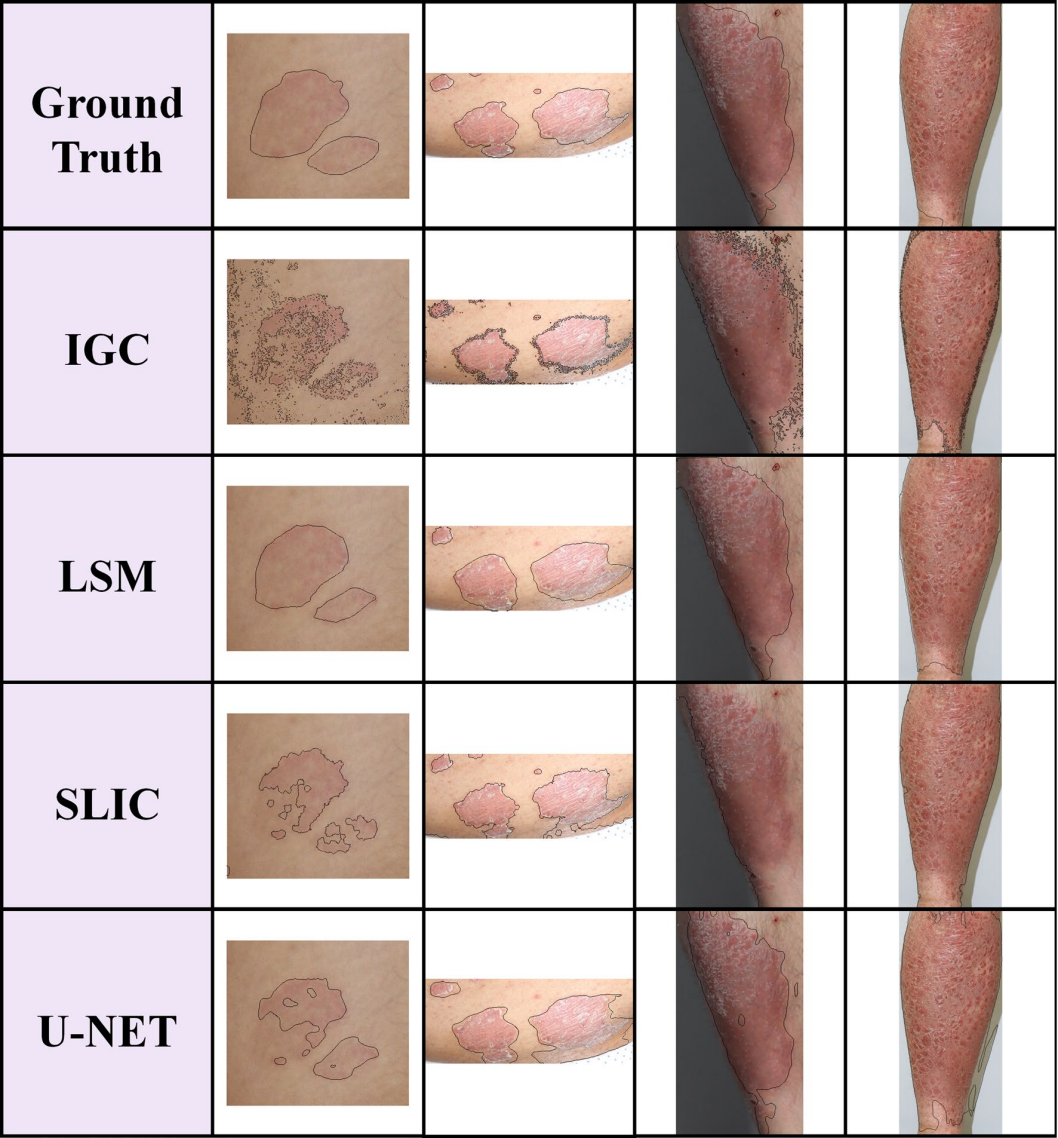
Psoriasis segmentation results of four segmentation methods, IGC, LSM, SLIC superpixels, and U-Net. The columns show psoriasis lesion images with different shapes, sizes, colors, and severities. It shows the segmentation results of IGC, LSM, SLIC superpixels, and U-Net, where the disease ambiguous or environmental noises such as shadows, lighting, etc., make accurate segmentation performance difficult.

Figure 2 shows the representative segmentation results of the four algorithms. There are four different types of psoriasis based on different locations, sizes, colors, and severity. In the initial stage of psoriasis, the shape of the disease boundary is ambiguous (1st column). It is difficult to determine the boundary due to body curvature, shading, and environmental noise (2nd and 3rd columns) or when psoriasis is too severe. The range of disease is expanding; it becomes difficult to distinguish the boundaries between the disease and the surrounding normal skin (4th column). These become factors that make accurate segmentation difficult^21^. All four algorithms generally perform accurate segmentation, even with challenging images. However, in IGC, LSM, which relies on subjective judgment, may differ in the selection of multiple disease regions depending on the segmentation performer and may take a long time. IGC performs segmentation while searching for pixels based on the seed points the user inputs directly; therefore, the results appear disordered for a boundary between the object region and the background. SLIC and the U-Net model cannot perform precise segmentation for psoriatic diseases located in areas containing body curvature, lighting, and shading.

Next, Table 1 shows the average value of each similarity index when we conduct a similarity evaluation of the ground truth (GT) and the results of each segmentation algorithm. We increased the reliability of the segmentation performance evaluation using four types of similarity evaluation indicators. It is possible to evaluate not only the overlap region with GT (dice similarity coefficient (DICE), global consistency error (GCE), variation of information (VoI), and Rand index (RI)) but also the spatial location of the segmentation region (Hausdorff distance (HD)). DICE, VoI, and RI indicate that the larger the value, the more similar the GT and the segmentation results. GCE and HD indicate that the smaller the value, the more similar the GT and segmentation results. LSM and SLIC superpixels (semi-automated and automated methods, respectively) showed the best performance. In particular, LSM shows the best similarity results for the DICE and RI indicators and SLIC superpixels for the VoI and HD indicators. We can analyze the disadvantages of each segmentation method based on the similarity results. The results of the LSM are similar to those of the GT because the users directly provided the seed points to the psoriasis area. Conversely, in IGC, the user also provided the seed points of the psoriasis region and background, but it greatly affected various noises such as body curvature and shadow. The processing time of the SLIC superpixels method is short; however, if the disease severity is low or very high, it cannot perceive the color difference between the psoriasis region and the unaffected region of the skin (normal); thus, its result tends to become over segmented. Segmentation by the U-Net model is difficult in cases where the size and color of the disease are ambiguous and for new types of psoriasis images that have not been learned. It can intuitively confirm the similarity value, as shown by the graphs in the supplementary data (Fig. S1). We can see that there is no significant difference in the segmentation performance of all algorithms. Despite their disadvantages, their segmentation results are similar to those of GT. We confirm that all segmentation algorithms can be used for the segmentation of the psoriasis region.

**Table 1:** Results of five similarity indicators comparing segmentation results of each algorithm and the ground truth.

Method		DICE	GCE	VoI	HD	RI
Semi-automated segmentation	IGC	0.936	**0.112**	0.989	**139.014**	0.869
	LSM	**0.945**	0.113	**1.158**	332.567	**0.882**
Automated segmentation	SLIC	**0.915**	0.149	**1.317**	**76.289**	**0.856**
	U-Net	0.911	**0.129**	1.038	332.583	0.822

The bold values indicate the best results.

When we evaluate the similarity of psoriasis segmentation images and GT, LSM is best in the semi-automated segmentation methods and SLIC superpixels in the automated segmentation methods.

*DICE* dice similarity coefficient, *GCE* global consistency error, *VoI* variation of information; *HD* Hausdorff distance; *RI* Rand index.

A good segmentation algorithm is not only accurate (which is the similarity between the segmentation result and the GT) but is also repeatable and reproducible (which can produce consistent segmentation results for psoriasis regions, including various shapes and environmental noises) and is efficient (which is capable of fast performance). In the case of semi-automatic algorithms, they can obtain more accurate segmentation results by including the user’s seed points, but the more disease areas within the observation range, the more ambiguous the boundary or shape of the disease and the greater the error in the subjective evaluation and the time required for the segmentation process. That is, semi-automatic algorithms are inefficient because they have a long execution time and low reproducibility. U-Net, an automatic segmentation algorithm, needs to perform fine-tuning considering the characteristics of psoriasis and learn various types of psoriasis to improve the performance of psoriasis segmentation.

### Patch image generation of the features of psoriasis

In psoriasis, the higher the severity, the more prominent the erythema and scaling characteristics. Among the evaluation indicators of the PASI score, erythema and scaling were characterized by a clear difference in color. The more severe the disease, the higher the color intensity (redness) of the erythema, and the scaling is stacked in layers and appears clear white. Erythema and scaling coincide in the same disease region. We used 18 out of 70 disease images and then obtained patch images of erythema and scales utilizing the color difference in the segmentation images. In this case, the segmentation image used is the resulting image based on the SLIC superpixels method, which has the best segmentation performance.

**Figure 3:**
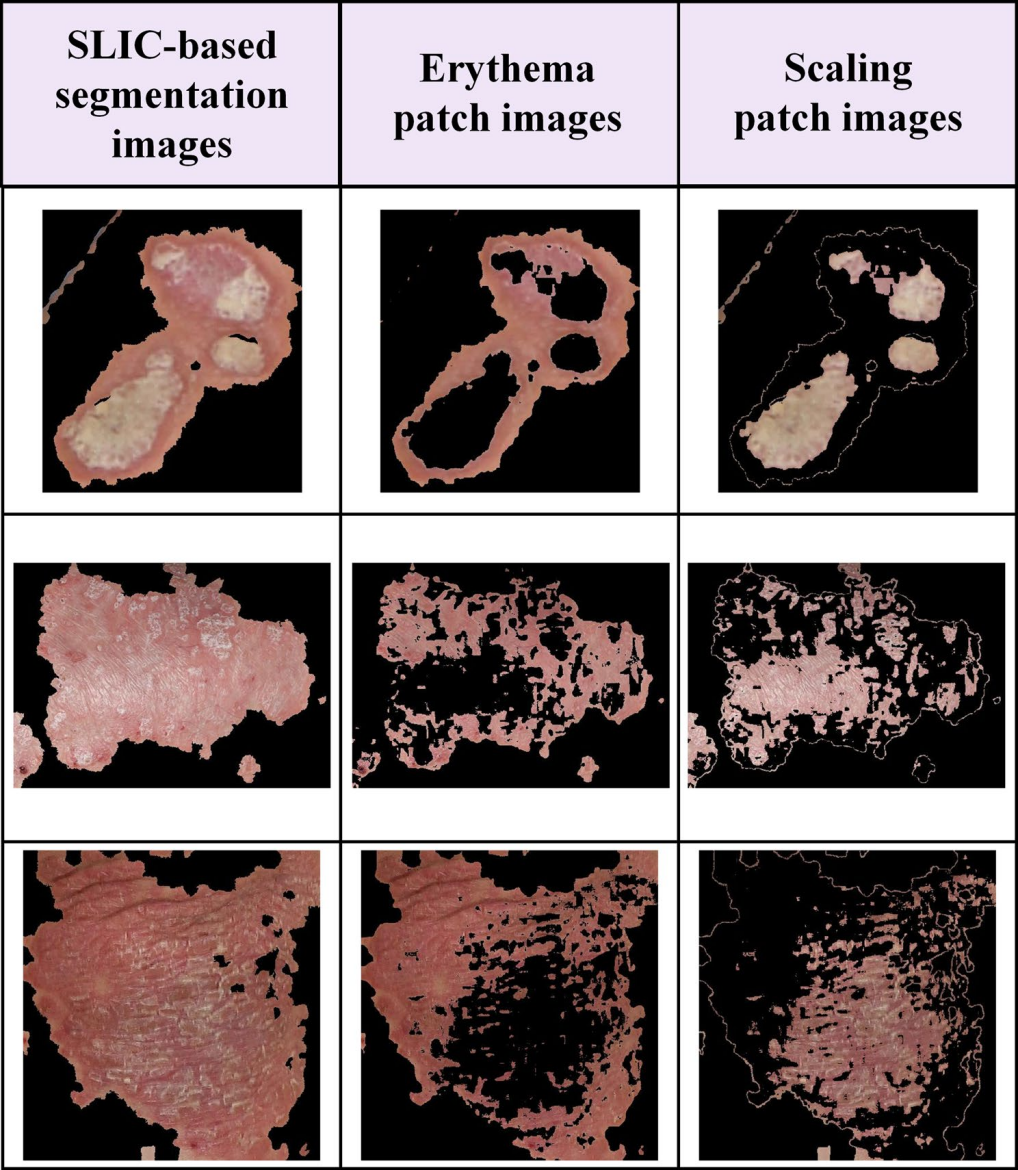
The patch images for erythema and scaling are generated from segmentation images of the SLIC superpixels method. This figure shows the representative segmentation images of the SLIC superpixels method and patch images for erythema and scaling that coincide with psoriasis.

To distinguish the two symptoms based on color features, we converted the RGB space of the disease segmentation image to CIE-L*a*b* space and performed k-means clustering to create patch images for erythema and scaling. Figure 3 shows patch images of erythema and scaling generated from the segmentation image. The first column is a segmentation image of the SLIC superpixels, the second column shows the patch images of erythema features, and the third column shows the patch image of scaling features. Since it is difficult to visually separate and evaluate erythema and scaling that coincide, it is possible to evaluate the severity of erythema and scaling with patch images. Patch images of erythema and scaling have distinct differences in color and texture depending on the severity of psoriasis. Hence, the generalization performance of psoriasis severity classifiers can be improved by using patch images.

### Classification severity of psoriasis

**Figure 4:**
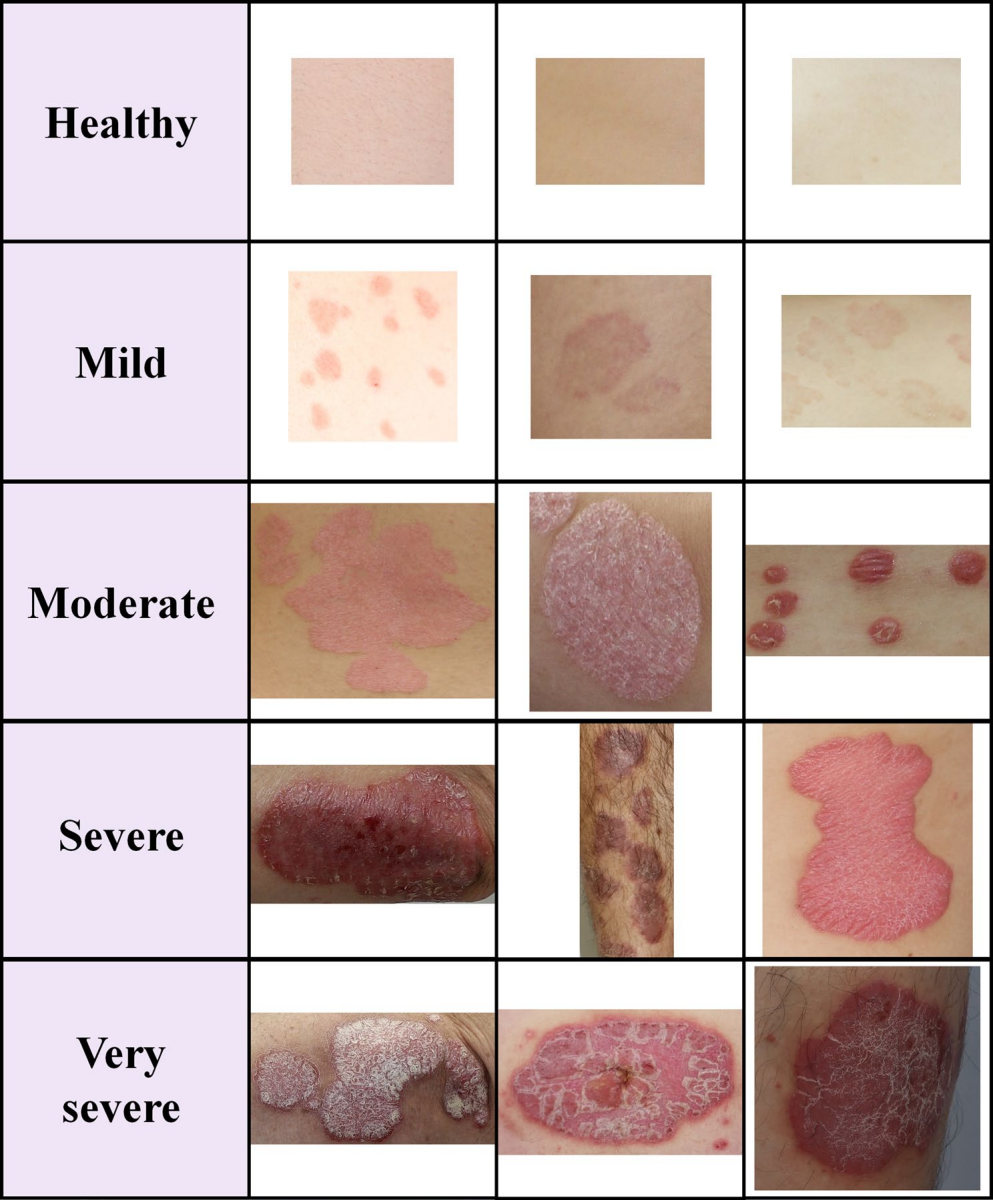
Representative images of psoriatic disease for a total of five severity groups. Healthy skin is normal skin, where no lesions exist. From mild to very severe, they are images classified based on the scoring value, which is the sum of PASI parameters (erythema, scaling), by the clinician. Images have random sizes owing to the body parts and lesion shape, size, etc.

In the severity classification, we divided 70 disease images into four groups (mild, moderate, severe, and very severe) according to the severity and used five groups of images, including healthy skin (10 images)^22^. Figure 4 shows the representative images of each group. We applied two data augmentation techniques to improve the classification performance, and generated classification datasets (Datasets A and B). We then performed a comparative evaluation of six representative classifiers for multiple classifications of psoriasis severity using the generated datasets.

For the optimization study of the severity classification of psoriasis, we performed classification performance evaluation of the six classifiers as well as an analysis of disease characteristics (texture, color, and spectrum) and data augmentation techniques that affect classification performance. First, to evaluate the relevance of the disease severity and the extracted features, we trained the classifiers using a single feature (three cases), a combination of features (three cases), or all the features (one case). Next, to analyze the difference in classification performance through data augmentation, we generated dataset A consisting of only crop images, and dataset B consisted of crop images and geometric transformation images (Table S1); the effect of data augmentation on the classification performance is then analyzed. Finally, we added patch images of erythema and scales to the datasets and investigated the utility and usefulness of patch images in the severity classification. Moreover, we present the results of the overall evaluation indicators for the performance of the classifiers for each dataset in the supplementary data (Table S2). We conducted k-fold cross-validation and evaluated the performance of the classifiers using seven evaluation indicators.

The psoriasis data were imbalanced. Hence, we used the F1-score to evaluate the classification performance. When comparing the classification results of each classifier according to the feature combination, Tables 2 and 3 show the results for Datasets A and B, respectively. For the classification results using Dataset A, DNN showed the best classification performance for texture, color, and combination of texture and color features, RF for spectrum and the combination of color and spectrum features, and k-NN for the combination of texture and spectrum and all the features. Overall, the classification results were the best when only the texture feature was used and the worst when only the spectrum feature was used. In addition, among the six classifiers, NB showed the worst performance, and k-NN generally showed the best performance in all feature combinations. In the case of a DNN, the performance deviation of classification depends on the feature combinations. Furthermore, Table 3 shows the classification results using Dataset B; DNN shows the best performance for texture features and RF for combining the remaining six types of features. Overall, the classification results were the best when the combination of texture and color features was used. As with the results in Dataset A, the classification results were the worst when only the spectrum feature was used. In addition, the classification performance of ECOC-SVM was the worst, and RF showed the best performance. Similarly, the classification results of the DNN showed accuracy deviations according to the types of feature combinations. The classification performance increased in most methods when the classifiers were trained using Dataset B rather than Dataset A.

**Table 2:** Results of features-based psoriasis severity classification in Dataset A.

Classifier	Texture	Color	Spectrum	Texture + Color	Texture + Spectrum	Color + Spectrum	Full
ECOC-SVM	0.742	0.625	0.573	0.726	0.639	0.614	0.627
NB	0.578	0.558	0.515	0.576	0.626	0.569	0.499
k-NN	0.914	0.609	0.732	0.867	**0.845**	0.796	**0.926**
RF	0.777	0.723	**0.756**	0.775	0.781	**0.799**	0.863
AB	0.755	0.668	0.719	0.736	0.737	0.747	0.868
DNN	**0.99**	**0.868**	0.713	**0.951**	0.681	0.683	0.779

The bold values indicate the best results.

It shows the F1-score comparison for the six classifiers performance according to the combination of features. We created several combinations using texture, color, and spectrum, and Full is the case of using all features.

**Table 3:** Results of features-based psoriasis severity classification in Dataset B.

Classifier	Texture	Color	Spectrum	Texture + Color	Texture + Spectrum	Color + Spectrum	Full
ECOC-SVM	0.769	0.666	0.517	0.821	0.585	0.575	0.821
NB	0.876	0.877	0.507	0.877	0.63	0.591	0.697
k-NN	0.954	0.985	0.816	0.95	0.897	0.932	0.9
RF	0.947	**0.989**	**0.84**	**0.99**	**0.939**	**0.98**	**0.986**
AB	0.791	0.928	0.636	0.961	0.8	0.916	0.934
DNN	**0.958**	0.951	0.597	0.97	0.596	0.62	0.93

The bold values indicate the best results.

It shows the F1-score comparison for the six classifiers performance according to the combination features. We created likewise several combinations using texture, color, and spectrum, and Full is the case of using all features.

Based on these results, we selected k-NN, RF, and DNN, which showed the best performance on datasets A and B, and evaluated their classification performance according to the four types of datasets (Tables 4, 5 and 6). Similarly, we compared the classification performance using the F1-score. We can confirm that the classification performance increases with the combination of features, including the color features of the dataset that includes patch images. When patch images were included in dataset B, the k-NN and RF models exhibited a very slight performance degradation of approximately 0.01, but the classification performance improved for the combination features of texture and color in the DNN models. Therefore, it can be confirmed that the patch images of erythema and scale have a clear color difference, which helps improve the color information-based psoriasis severity classification performance. Overall, we obtained the best performance with dataset B only. However, we expect to improve the generalization performance of the classifier by using more patch images.

**Table 4:** Classification results of the k-NN model according to dataset types.

Parameters	Dataset A only	Dataset A + patch images	Dataset B only	Dataset B + patch images
Texture	0.908	0.871	0.954	0.94
Color	0.609	0.622	0.985	0.972
Spectrum	0.732	0.698	0.816	0.811
Texture + color	0.867	0.874	0.95	0.926
Texture + spectrum	0.875	0.798	0.897	0.890
Color + spectrum	0.796	0.817	0.932	0.917
Full	0.926	0.819	0.921	0.932

It shows the F1-score of the performance of the k-NN model. We trained the classifier using combinations of features extracted from the new dataset (including patch images) or the initial dataset and identified the difference in the classification performance of the k-NN model.

**Table 5:** Classification results of the RF model according to dataset types.

Parameters	Dataset A only	Dataset A + patch images	Dataset B only	Dataset B + patch images
Texture	0.77	0.784	0.947	0.934
Color	0.609	0.666	0.989	0.979
Spectrum	0.756	0.715	0.84	0.828
Texture + color	0.775	0.796	0.99	0.984
Texture + spectrum	0.781	0.823	0.939	0.932
Color + spectrum	0.799	0.786	0.98	0.97
Full	0.863	0.837	0.989	0.978

It shows the F1-score of the performance of the RF model. We trained the classifier using combinations of features extracted from the new dataset (including patch images) or the initial dataset and identified the difference of classification performance of the RF model.

**Table 6:** Classification results of a DNN model according to dataset types.

Parameters	Dataset A only	Dataset A + patch images	Dataset B only	Dataset B + patch images
Texture	0.991	0.909	0.958	0.916
Color	0.868	0.966	0.951	0.942
Spectrum	0.713	0.739	0.597	0.526
Texture + color	0.951	0.932	0.97	0.989
Texture + spectrum	0.681	0.64	0.596	0.564
Color + spectrum	0.683	Nan	0.62	0.602
Full	0.779	0.765	0.945	0.974

It shows the F1-score of the performance of a DNN model. We trained the classifier using combinations of features extracted from the new dataset (including patch images) or the initial dataset and can identified the difference of classification performance of a DNN model.

**Figure 5:**
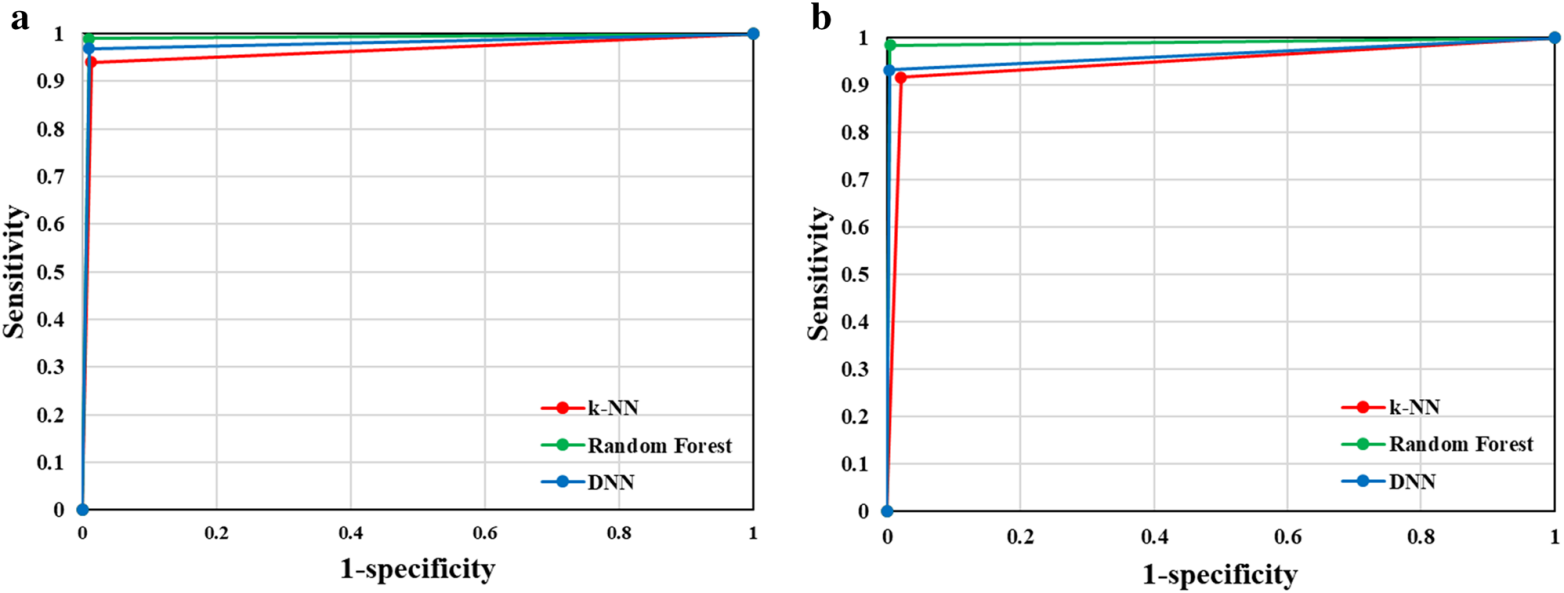
AUC graphs for psoriasis severity classification performance of k-NN, RF, and DNN model for different combinations of features or datasets. The three classifiers were commonly trained using a combination of texture and color features: (a) used only dataset B, and (b) used a new dataset B, including patch images.

Finally, we show the performance of the three classifiers as an area under the curve (AUC) graph (Fig. 5). Three classifiers were trained using a combination of texture and color features in dataset B or new dataset B, which included patch images, where this condition was shown to have the best classification performance. Figure 5a,b show the results for dataset B and the new dataset B with patch images, respectively. The graph confirms the classification performance in the order RF > DNN > k-NN, and all three classifiers classify psoriasis severity very well.

## Discussion

Currently, the PASI score is typically used for the diagnosis and evaluation of psoriatic diseases, but this evaluation index includes the subjective opinion of the clinician; therefore, there is a disadvantage in that the evaluation result is highly variable and has poor reproducibility^23^. To objectify the PASI score, various studies have conducted the segmentation of disease regions and severity classification within the observation range. A clear area where a disease appears is first detected, and a severity assessment is performed based on the detected disease area to diagnose psoriasis accurately. In other words, segmentation and classification studies should be performed simultaneously to diagnose and evaluate diseases. However, existing studies have conducted single segmentation and classification studies. Alternatively, research mainly conducted on the case where the used psoriasis image exists as a single disease within the observation range or the color and shape are clear, and the generalization performance of the previously proposed methods is limited. To compensate for these shortcomings, this study proposes methods optimal as a single study rather than independent studies of disease segmentation and classifiers for the accurate evaluation of psoriasis based on severity. The difference from the existing approaches for the segmentation and classification of psoriasis is that we used and analyzed the characteristics of simultaneous psoriasis symptoms and trained them on a multi-label classifier. In addition, a new dataset on psoriasis was developed to improve the generalization performance of psoriasis severity classifiers.

First, we used four segmentation methods (IGC^13,14^, LSM^15,16^, SLIC superpixels^11,17^, and U-Net^10,18^) and compared segmented psoriasis areas. These methods have been used for image segmentation in various fields. Especially SLIC, not only in existing psoriasis studies but also in various medical image segmentation studies. These methods were largely separated into semi-automatic and automatic algorithms and evaluated. The results of the LSM-based segmentation method in the semi-automatic segmentation algorithms and the SLIC-based segmentation method in the automatic algorithms showed high similarity with the GT. However, the semi-automatic segmentation algorithm has the disadvantage of low efficiencies, such as ambiguity of psoriatic disease and performance time according to the distribution of disease, and poor reproducibility of repetitive results due to subjective evaluation. Conversely, in an automatic algorithm, errors are generated due to body curvature, contrast, and ambiguity of the disease. In the SLIC-based segmentation method, when the distribution of psoriatic disease is high within the observation range, over-segmentation occurs, making it difficult to distinguish the presence or absence of the disease by visual observation and can be interpreted as the process of disease occurrence. The difference in performance between the four segmentation methods was not significant, and all four methods showed similar results to the GT, confirming their usefulness in segmenting psoriatic diseases. However, we selected the SLIC method (an automatic algorithm) as the psoriasis segmentation algorithm, taking into consideration time efficiency and reproducibility, and patch images for erythema and scale were generated using the segmentation results.

The appearance of the disease in patients with psoriasis varies in shape and distribution depending on their severity, and erythema and scales corresponding to the PASI score indicator vary; they include surrounding environmental noise such as body curvature, skin hairs, and shadows. Moreover, erythema and scales coincide, and in particular, it isn't easy to assess the degree of erythema by visual observation because of the thickness of the scales. Therefore, this study deals with images that have various forms depending on the severity and have various psoriasis symptoms within the observation range. Furthermore, two-dimensional images of random psoriasis are considered for the rest of the body parts, except the head, without distinction between parts of the body. This was not considered in this study because a three-dimensional analysis was required to evaluate the stiffness or thickness of the lesion area.

This study not only analyzed the results of the segmentation of psoriasis but also generated patch images for erythema and scales detected simultaneously in the segmented image and using the segmented image to attempt severity classification. For this new dataset on psoriasis, we trained multi-level classifiers to determine the severity of psoriasis. Multi-level classifiers are also lacking in research on classifier performance, which is best in evaluating psoriasis severity. In particular, for the dataset used in this study, the patch image may be a factor that can lower the performance of the classifier because of limited information regarding the image. However, the generalization ability of the classifier can be improved by learning the patch image. Therefore, we trained six multi-level classifiers on the new dataset and presented an optimal classifier that performs an accurate classification. The classifiers learned about a single feature or a combination of features extracted from psoriasis images; thus, they also analyzed features that are related deeply to disease severity. Furthermore, two types of data augmentation were applied to overcome a limited number of images, and we compared the effect of two types of datasets. As a result, out of the six classifiers, k-NN, RF, and DNN showed good classification performance. In particular, when using the combination of texture and color features extracted from dataset B constructed through three methods, the classification performance showed 95% or more good results. In addition, when the classifier was trained using a combination of color features from the result of the dataset combining dataset A and the patch images, the classification performance increased compared to that using only dataset A. Based on these results, differences in the characteristics of erythema and scales according to the severity were confirmed, and it was verified that the severity classification for the psoriatic disease was more effective through patch images. Additionally, it was confirmed that two types of datasets could influence classification performance, that is, data augmentation techniques regarding whether affine transforms. In other words, the number of psoriasis images in our study was small, but we confirmed that good classification performance could be derived by combining the applied data augmentation and patch images for psoriasis symptoms.

We constructed a novel dataset on psoriasis for the segmentation and severity classification of psoriasis lesions by collecting images directly from patients who received psoriasis treatment at the hospital. Since psoriasis symptoms are distributed throughout the body, clinical diagnosis of psoriasis is performed by dividing the whole body into sections and calculating the score for each evaluation parameter of the PASI score to determine the severity. The images are parts of the entire body. In actual clinical practice, the medical examiner generally calculates the PASI score based on the area where psoriasis is the most severe relative to the various body compartment images. However, the dataset on psoriasis constructed in this study and the existing datasets on psoriasis is mainly composed of locally cropped images. We confirmed that the image-based PASI score of the whole body and the local image-based PASI score was partially different even though the images were collected from the same patient during the experiment. These results caused a deviation in the severity based on the actual PASI criteria of this study and the severity score predicted by the optimal classifier. The reason for this deviation is that it is difficult to consider the area or induration in the local image. Therefore, based on these results, we reevaluated the PASI score for the newly created local image of psoriasis and then conducted a classification study. Therefore, a future research on PASI parameters that require three-dimensional information, such as area and thickness, considering various skin colors and psoriatic diseases that appear throughout the body is needed^24^. Furthermore, skin diseases such as eczema, tinea corporis, and atopy are difficult to distinguish without the help of experts because of similar symptoms such as redness and itching. In the future, we will study a solution for diagnosing psoriasis among skin diseases with similar symptoms using images of control skin disease to study diagnostic systems for psoriasis.

## Methods

### Data acquisition and building

In this study, to conduct research using psoriasis image data collected directly from the clinic, the participating institutions, Korea University Guro Hospital and Soonchunhyang University, were approved according to the guidelines of the Institutional Review Board of each institution, and the research was conducted in accordance with the ethical principles of the Declaration of Helsinki. (Approval number: 2020GR0019, 202001-BM-005). We recruited 35 Korean patients with psoriasis in the clinic and obtained written informed consent. Among the whole-body data, head images were excluded from this study^25^. Since this study deals with 2D images of the local disease area, dermatologists assigned 0–4 points according to severity for the characteristics of erythema and scales based on local disease images. These psoriasis images and severity scores for erythema and scale were transferred from researchers at clinical institutions to researchers at Soonchunhyang University to conduct further studies on psoriasis. We used 80 acquired local disease images for the segmentation and classification studies. For classification, we divided images into five severity groups, and applied a data augmentation technique to improve classification performance. For the psoriatic disease image, the dermatologist assigned 0–4 points for erythema and scaling according to the severity and divided the disease severity group based on the sum of the scores of the two evaluation indicators. The severity of psoriasis was divided into five groups: healthy, mild (1–2 points), moderate (3–4 points), severe (5–6 points), and very severe (7–8 points).

### Pre-processing and data augmentation

The psoriasis image includes features of psoriasis, such as red erythema and white scales. Because of the psoriasis image used in this study, it is difficult to obtain images of various severities sufficiently. Therefore, data augmentation was used to overcome the problem of the number of images. Data augmentation made it possible to consider the invariability of image features generated through various transformations, enabling a more robust algorithm optimization. Furthermore, because the unique characteristics of the image expressed in the data can be efficiently reflected, the prediction range can be slightly widened, and overfitting can be prevented^18^. In addition, the classification performance may vary depending on the augmentation technique; therefore, a comparative study on the effective data augmentation method for improving the performance is needed. In this study, two types of data augmentation were performed, and a comparative evaluation was performed on the dataset, which led to the excellent performance of the classifier. The first method was to crop the local image of psoriasis on a certain basis^19^. The images were cropped into six or four parts based on the size of the image so that the psoriasis area could be well included. At this time, the size of the cropped images and the original images were random and not uniform. The second method consists of the crop process and the affine transform technique such as rotation, flipped, and scaling^19,26,27^. The rotation was changed by 90°, 180°, and 270°, and then flipped. Scaling was used for expansion, reduction, horizontal stretching, and vertical stretching. After performing two data augmentations, the final psoriatic disease dataset was constructed by excluding the case where no disease was included, or the disease region was too small as a post-processing step.

In this study, we extracted a patch image that included information on erythema and scale. Because these two features appear simultaneously, it is difficult to distinguish them with the naked eye; therefore, we tried to objectify each feature by generating a patch image. The best segmentation method among psoriatic disease segmentation methods was transformed into CIE Lab space, and then color information-based k-means clustering was performed to generate patch images for erythema and scales. Additional data augmentation was not applied to the newly developed patch image in the disease segmentation image because it contained limited information. We also presented the number of each severity group image used for classifier training after data augmentation (Table S2).

### Experimental setup

We conducted this study using MATLAB R2020b (MathWorks, Inc., Natick, MA, USA. Parallel computing was performed for classifier learning. To evaluate the image segmentation algorithm, two researchers in charge of the study produced GT after receiving training on the criteria for the diagnosis of psoriasis by a dermatologist. GT was used to analyze the similarity of the results of the segmentation algorithm.

### Psoriasis lesion segmentation method

Determining the severity of psoriasis is very difficult with the naked eye owing to the disease variety. We conducted a comparative study of semi-automatic and automatic segmentation algorithms.

First, when the user scribes the initial seed on the foreground and background in the image, the IGC segments the foreground and background by finding a globally optimized segmentation while minimizing energy from the initial seed^13,14^. The variable value set for the psoriasis lesion segmentation was specified as a mask with 5 pixels, and 16 bins per channel in the case of bins were fixed to perform segmentation of the entire image.

LSM is an active contour method^15,16^. In this study, a response-diffusion term was applied to improve the image segmentation speed and operate flexibly to changes in topology to express a clear segmentation boundary. Among the variables of the RD-LSM used in this study, the fixed constants were fixed at $\Delta {t}_{1}=1$, $\Delta {t}_{2}=.001$, and the iteration was 15.

The SLIC superpixels-based segmentation method generates an over segmented image based on a 5-dimensional feature space [L*, a*, b*, x, y] defined by the CIE-L*a*b* color space and pixel coordinates (x, y) from an RGB image and segments a disease region through local k-means clustering^11,17^. At this time, the number of superpixels is 700, the number of center points is three, and the number of iterations is 500.

U-Net is a model proposed for image segmentation in the biomedical field^10^. We retrained our dataset on psoriasis using a U-Net model trained with cell images provided by Ronneberger^18^ and tested it using different datasets on psoriasis (70 images) to obtain a predicted probability map. To train the dataset, the batch size was set to two, and the epoch was set to 850.

### Similarity analysis

To evaluate the performance of the four segmentation algorithms, we used five similarity indicators to compare the results of segmentation and GT. The overlap-based similarity indicators DICE^28^ and GCE^29^, information-based similarity indicators VoI^29^, spatial distance-based similarity indicators HD^30^, and pair-counting-based similarity indicator RI^29^ are used. The higher the DICE, VoI, and RI values and the lower the HD and GCE, the more similar the GT.

### Psoriasis feature extraction and selection

We extracted texture features (gray-level co-occurrence matrix^31^, gray-level run-length matrix, intensity histogram, semi-variogram^19^, and local binary patterns^32^), color features (RGB, HSV, CIE-L*a*b*, and YCbCr), and spectrum features (Gabor filter and Zernike moment^33^).

We performed feature selection to improve the reliability, safety, and classification accuracy of the psoriasis severity classification models^12,34^. We selected important features using the filter method, which utilizes feature characteristics such as the variation and relevance of features. We can obtain ranking information of important features by using statistical methods, the minimum redundancy maximum relevance (mRMR) algorithm^35^, the chi-square test, and the ReliefF algorithm^36^. We create three feature subsets through each method by selecting 2/3 of all the features with a high ranking and then generate a final feature set by selecting only features that are duplicated in these three feature subsets.

As a result, we generated texture features (67), color features (27), spectrum features (62), and full features (160) for dataset A, and texture features (75), color features (25), spectrum features (56), and full features (156) for dataset B.

### Psoriasis severity classification model

In this study, we compared and evaluated six classifiers to perform multi-label classification of psoriasis severity.

The ECOC-SVM model is an ECOC model that solves multi-label classification tasks by combining a binary SVM classifier using a code matrix^37^. It has the disadvantage of requiring a long time.

k-NN is a feature-based classification model that classifies new data based on k center points and uses only data information without a separate model generation process. Therefore, its performance time is short but vulnerable to noise.

Naïve Bayes is a simple supervised learning classifier based on Bayes’ theory. It performs classification by assuming independence between the characteristics of data, and it is a method that has been widely used in the medical field as well as text classification.

Random Forest (RF) and AdaBoosting are ensemble learning-based classifiers. We created an RF model by bagging 100 decision trees as weak classifiers and an Adaboosting model by boosting. The ensemble method not only improves the classification performance, but also prevents overfitting of the models compared to using a single classifier.

Deep neural networks (DNNs) are artificial intelligence-based models. We created a DNN classifier composed of four fully connected layers. The data batch size was 32, the loss function was cross-entropy, and the learning rate was 0.001. Adam was used to optimizing the model parameters, and the epoch was 200. The hyperparameters of the model were empirically set.

To equally evaluate the performance of machine learning and deep learning classifiers, each classifier performs k-fold cross-validation ten times. We then calculated the accuracy, sensitivity, specificity, precision, F1-score, Matthews correlation coefficient (MCC), and kappa coefficient to evaluate the classification performance. The F1-score, MCC, and kappa coefficient are indicators that consider the imbalance of the dataset.

## Supplementary Information

Supplementary Information.
