# Supplementary material for: Optimization of psoriasis assessment system based on patch images
Source: Sci Rep. 2021 Sep 13;11:18130. doi: 10.1038/s41598-021-97211-9 (PMC8437948; doi:10.1038/s41598-021-97211-9)
Supplement: Supplementary file 1 — Supplementary Information. [file 41598_2021_97211_MOESM1_ESM.docx]

***Figure S1. Similarity graphs between the ground truth and four segmentation methods.***

***
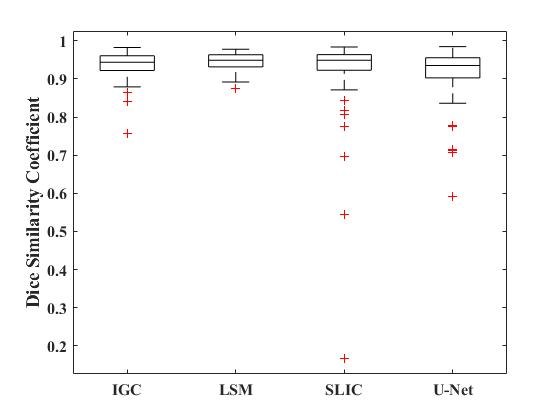
***

***
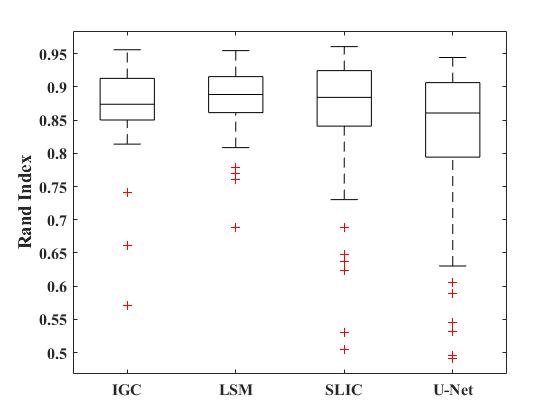

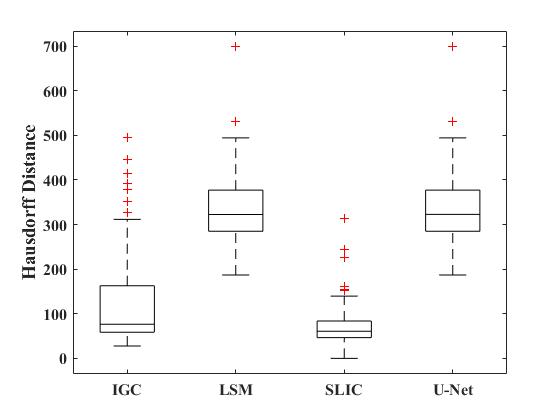

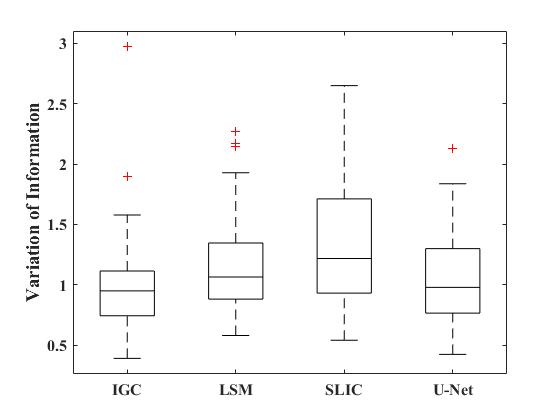

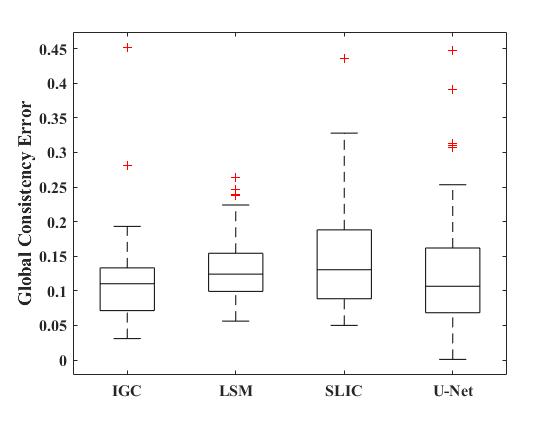
***

***Table S1. The number of images in severity group according to data augmentation.***

| **Number**  **Group** | **Original**  **images** | **Dataset A**  (Crop only) | **Dataset B**  (Crop, Rotate, Scale) | **Patch images**  **(erythema + scaling)** |
| --- | --- | --- | --- | --- |
| **Healthy** | 10 | 50 | 330 | - |
| **Mild** | 16 | 83 | 546 | 2 |
| **Moderate** | 22 | 115 | 774 | 19 |
| **Severe** | 22 | 111 | 741 | 25 |
| **Very severe** | 10 | 61 | 427 | 10 |

***Table S2. Results of multiple classifiers performance.***

***Dataset A***

1. ***Texture parameters***

| **Classifier** | **Accuracy** | **Sensitivity** | **Specificity** | **Precision** | **F1-score** | **MCC** | **Kappa** |
| --- | --- | --- | --- | --- | --- | --- | --- |
| **ECOC-SVM** | 0.7119 | 0.7442 | 0.9241 | 0.7402 | 0.742 | 0.6662 | 0.0997 |
| **NB** | 0.5667 | 0.6139 | 0.8888 | 0.5778 | 0.5887 | 0.4840 | 0.2615 |
| **k-NN** | 0.90 | 0.9046 | 0.9736 | 0.9139 | 0.9077 | 0.8826 | 0.6875 |
| **RF** | 0.7571 | 0.7648 | 0.9362 | 0.7765 | 0.7698 | 0.7070 | 0.2411 |
| **AB** | 0.7238 | 0.7272 | 0.9270 | 0.7545 | 0.7369 | 0.6672 | 0.1369 |
| **DNN** | 0.9905 | 0.9922 | 0.9976 | 0.9896 | 0.9909 | 0.9884 | 0.9702 |

1. ***Color parameters***

| **Classifier** | **Accuracy** | **Sensitivity** | **Specificity** | **Precision** | **F1-score** | **MCC** | **Kappa** |
| --- | --- | --- | --- | --- | --- | --- | --- |
| **ECOC-SVM** | 0.5905 | 0.6258 | 0.8924 | 0.6267 | 0.6251 | 0.5185 | 0.2186 |
| **NB** | 0.5238 | 0.5631 | 0.8758 | 0.5547 | 0.5579 | 0.4346 | 0.3280 |
| **k-NN** | 0.581 | 0.6127 | 0.8901 | 0.6098 | 0.6093 | 0.5010 | 0.2364 |
| **RF** | 0.6952 | 0.7222 | 0.9198 | 0.7245 | 0.7229 | 0.6431 | 0.0476 |
| **AB** | 0.6357 | 0.6669 | 0.9043 | 0.6699 | 0.6682 | 0.5726 | 0.1216 |
| **DNN** | 0.8381 | 0.8621 | 0.9561 | 0.8809 | 0.8680 | 0.8272 | 0.4940 |

1. ***Spectrum parameters***

| **Classifier** | **Accuracy** | **Sensitivity** | **Specificity** | **Precision** | **F1-score** | **MCC** | **Kappa** |
| --- | --- | --- | --- | --- | --- | --- | --- |
| **ECOC-SVM** | 0.5643 | 0.5726 | 0.8853 | 0.5904 | 0.5731 | 0.4651 | 0.2656 |
| **NB** | 0.5024 | 0.5305 | 0.8721 | 0.5075 | 0.5149 | 0.3898 | 0.3569 |
| **k-NN** | 0.719 | 0.7304 | 0.9266 | 0.7358 | 0.7322 | 0.6597 | 0.122 |
| **RF** | 0.7405 | 0.7444 | 0.9303 | 0.8007 | 0.7562 | 0.6984 | 0.1890 |
| **AB** | 0.719 | 0.7138 | 0.9257 | 0.7404 | 0.7193 | 0.6515 | 0.1220 |
| **DNN** | 0.6976 | 0.7040 | 0.9198 | 0.7844 | 0.7125 | 0.6593 | 0.0551 |

1. ***Combining of texture and color parameters***

| **Classifier** | **Accuracy** | **Sensitivity** | **Specificity** | **Precision** | **F1-score** | **MCC** | **Kappa** |
| --- | --- | --- | --- | --- | --- | --- | --- |
| **ECOC-SVM** | 0.7976 | 0.728 | 0.9515 | 0.7291 | 0.7264 | 0.6797 | 0.3676 |
| **NB** | 0.6971 | 0.6366 | 0.9302 | 0.5858 | 0.5755 | 0.5308 | 0.0534 |
| **k-NN** | 0.9021 | 0.8692 | 0.9765 | 0.866 | 0.8667 | 0.844 | 0.6941 |
| **RF** | 0.8333 | 0.7676 | 0.9595 | 0.7859 | 0.7746 | 0.7358 | 0.4792 |
| **AB** | 0.8056 | 0.7308 | 0.9534 | 0.7452 | 0.7361 | 0.6908 | 0.3924 |
| **DNN** | 0.9381 | 0.9534 | 0.9834 | 0.954 | 0.9509 | 0.9367 | 0.8065 |

1. ***Combining of texture and spectrum parameters***

| **Classifier** | **Accuracy** | **Sensitivity** | **Specificity** | **Precision** | **F1-score** | **MCC** | **Kappa** |
| --- | --- | --- | --- | --- | --- | --- | --- |
| **ECOC-SVM** | 0.7394 | 0.6331 | 0.9371 | 0.6459 | 0.6387 | 0.5769 | 0.1857 |
| **NB** | 0.7249 | 0.6331 | 0.9339 | 0.6284 | 0.6257 | 0.5628 | 0.1402 |
| **k-NN** | 0.9048 | 0.8712 | 0.977 | 0.8788 | 0.8745 | 0.8519 | 0.7024 |
| **RF** | 0.8413 | 0.7673 | 0.9609 | 0.8025 | 0.7806 | 0.7449 | 0.504 |
| **AB** | 0.8082 | 0.7233 | 0.9532 | 0.7617 | 0.7371 | 0.6942 | 0.4006 |
| **DNN** | 0.6714 | 0.6726 | 0.913 | 0.7804 | 0.6806 | 0.6329 | 0.0261 |

1. ***Combining of color and spectrum parameters***

| **Classifier** | **Accuracy** | **Sensitivity** | **Specificity** | **Precision** | **F1-score** | **MCC** | **Kappa** |
| --- | --- | --- | --- | --- | --- | --- | --- |
| **ECOC-SVM** | 0.7249 | 0.6129 | 0.934 | 0.6179 | 0.6138 | 0.5491 | 0.1402 |
| **NB** | 0.6878 | 0.5847 | 0.926 | 0.5835 | 0.5692 | 0.5055 | 0.0245 |
| **k-NN** | 0.8492 | 0.7965 | 0.9636 | 0.7963 | 0.796 | 0.7602 | 0.5288 |
| **RF** | 0.8558 | 0.7916 | 0.9653 | 0.8105 | 0.7985 | 0.7656 | 0.5494 |
| **AB** | 0.8175 | 0.7481 | 0.9566 | 0.746 | 0.7466 | 0.7032 | 0.4296 |
| **DNN** | 0.6714 | 0.6463 | 0.9109 | 0.8171 | 0.6826 | 0.6387 | 0.0261 |

1. ***Full parameters***

| **Classifier** | **Accuracy** | **Sensitivity** | **Specificity** | **Precision** | **F1-score** | **MCC** | **Kappa** |
| --- | --- | --- | --- | --- | --- | --- | --- |
| **ECOC-SVM** | 0.6048 | 0.6289 | 0.8974 | 0.6264 | 0.6273 | 0.5249 | 0.1904 |
| **NB** | 0.4786 | 0.5093 | 0.8655 | 0.4914 | 0.4992 | 0.3655 | 0.3863 |
| **k-NN** | 0.9238 | 0.9239 | 0.9801 | 0.93 | 0.9264 | 0.9069 | 0.7619 |
| **RF** | 0.8476 | 0.8617 | 0.9597 | 0.8649 | 0.863 | 0.8231 | 0.5238 |
| **AB** | 0.8524 | 0.8633 | 0.9608 | 0.8729 | 0.8676 | 0.8289 | 0.5387 |
| **DNN** | 0.7643 | 0.7781 | 0.9378 | 0.8058 | 0.7788 | 0.7258 | 0.2634 |

***Dataset B***

1. ***Texture parameters***

| **Classifier** | **Accuracy** | **Sensitivity** | **Specificity** | **Precision** | **F1-score** | **MCC** | **Kappa** |
| --- | --- | --- | --- | --- | --- | --- | --- |
| **ECOC-SVM** | 0.7449 | 0.7724 | 0.9332 | 0.7653 | 0.7686 | 0.7019 | 0.2027 |
| **NB** | 0.8655 | 0.8694 | 0.9643 | 0.8855 | 0.8764 | 0.8418 | 0.5797 |
| **k-NN** | 0.9571 | 0.9460 | 0.9885 | 0.9654 | 0.9542 | 0.9441 | 0.8658 |
| **RF** | 0.9411 | 0.9467 | 0.9845 | 0.9469 | 0.9467 | 0.9313 | 0.8159 |
| **AB** | 0.7661 | 0.7864 | 0.9381 | 0.7997 | 0.7914 | 0.7311 | 0.2692 |
| **DNN** | 0.956 | 0.957 | 0.9883 | 0.9628 | 0.958 | 0.948 | 0.8626 |

1. ***Color parameters***

| **Classifier** | **Accuracy** | **Sensitivity** | **Specificity** | **Precision** | **F1-score** | **MCC** | **Kappa** |
| --- | --- | --- | --- | --- | --- | --- | --- |
| **ECOC-SVM** | 0.6217 | 0.6721 | 0.9007 | 0.6618 | 0.6659 | 0.5676 | 0.1541 |
| **NB** | 0.8609 | 0.8672 | 0.9626 | 0.8947 | 0.8773 | 0.8437 | 0.5653 |
| **k-NN** | 0.9844 | 0.9844 | 0.9960 | 0.9847 | 0.9845 | 0.9805 | 0.9512 |
| **RF** | 0.9876 | 0.9886 | 0.9967 | 0.9892 | 0.9889 | 0.9856 | 0.9612 |
| **AB** | 0.9163 | 0.9284 | 0.9777 | 0.9282 | 0.9282 | 0.9060 | 0.7383 |
| **DNN** | 0.9368 | 0.9496 | 0.9829 | 0.9541 | 0.9506 | 0.9347 | 0.8026 |

1. ***Spectrum parameters***

| **Classifier** | **Accuracy** | **Sensitivity** | **Specificity** | **Precision** | **F1-score** | **MCC** | **Kappa** |
| --- | --- | --- | --- | --- | --- | --- | --- |
| **ECOC-SVM** | 0.4996 | 0.5118 | 0.8675 | 0.5398 | 0.5171 | 0.3916 | 0.3605 |
| **NB** | 0.4869 | 0.5299 | 0.8678 | 0.5133 | 0.5065 | 0.3879 | 0.3764 |
| **k-NN** | 0.8052 | 0.8146 | 0.9492 | 0.8178 | 0.8155 | 0.7653 | 0.3912 |
| **RF** | 0.83 | 0.8322 | 0.9547 | 0.8579 | 0.8397 | 0.7988 | 0.4688 |
| **AB** | 0.6249 | 0.6281 | 0.8992 | 0.6929 | 0.6363 | 0.553 | 0.1469 |
| **DNN** | 0.5972 | 0.5504 | 0.8896 | 0.8328 | 0.5973 | 0.5634 | 0.2055 |

1. ***Combining of texture and color parameters***

| **Classifier** | **Accuracy** | **Sensitivity** | **Specificity** | **Precision** | **F1-score** | **MCC** | **Kappa** |
| --- | --- | --- | --- | --- | --- | --- | --- |
| **ECOC-SVM** | 0.7952 | 0.8232 | 0.9459 | 0.8213 | 0.8210 | 0.7680 | 0.3601 |
| **NB** | 0.8591 | 0.8712 | 0.96204 | 0.8861 | 0.8770 | 0.8411 | 0.5597 |
| **k-NN** | 0.9507 | 0.9402 | 0.9866 | 0.9631 | 0.9498 | 0.9382 | 0.8459 |
| **RF** | 0.9879 | 0.9895 | 0.9968 | 0.9895 | 0.9895 | 0.9863 | 0.9623 |
| **AB** | 0.9549 | 0.9602 | 00.9880 | 0.9627 | 0.9614 | 0.9494 | 0.8592 |
| **DNN** | 0.9631 | 0.9685 | 0.99 | 0.9723 | 0.9701 | 0.9604 | 0.8847 |

1. ***Combining of texture and spectrum parameters***

| **Classifier** | **Accuracy** | **Sensitivity** | **Specificity** | **Precision** | **F1-score** | **MCC** | **Kappa** |
| --- | --- | --- | --- | --- | --- | --- | --- |
| **ECOC-SVM** | 0.5617 | 0.5872 | 0.8855 | 0.5866 | 0.5851 | 0.4723 | 0.2698 |
| **NB** | 0.6043 | 0.6334 | 0.8963 | 0.6318 | 0.6301 | 0.5284 | 0.1912 |
| **k-NN** | 0.8911 | 0.8869 | 0.9710 | 0.9089 | 0.8965 | 0.8688 | 0.6596 |
| **RF** | 0.9319 | 0.9377 | 0.982 | 0.9405 | 0.9390 | 0.9212 | 0.7871 |
| **AB** | 0.7732 | 0.7967 | 0.94 | 0.8031 | 0.7996 | 0.7399 | 0.2914 |
| **DNN** | 0.5919 | 0.6066 | 0.8965 | 0.6748 | 0.5964 | 0.5254 | 0.2159 |

1. ***Combining of color and spectrum parameters***

| **Classifier** | **Accuracy** | **Sensitivity** | **Specificity** | **Precision** | **F1-score** | **MCC** | **Kappa** |
| --- | --- | --- | --- | --- | --- | --- | --- |
| **ECOC-SVM** | 0.5518 | 0.5705 | 0.8817 | 0.5965 | 0.5747 | 0.4633 | 0.2860 |
| **NB** | 0.5688 | 0.6128 | 0.8877 | 0.5885 | 0.5906 | 0.4877 | 0.2578 |
| **k-NN** | 0.9223 | 0.9322 | 0.9795 | 0.9326 | 0.9321 | 0.9118 | 0.7571 |
| **RF** | 0.9773 | 0.9803 | 0.9940 | 0.98 | 0.9801 | 0.9742 | 0.9290 |
| **AB** | 0.9010 | 0.9126 | 0.9735 | 0.9193 | 0.9156 | 0.8895 | 0.6906 |
| **DNN** | 0.6299 | 0.5933 | 0.9013 | 0.752 | 0.6202 | 0.5605 | 0.1354 |

1. ***Full parameters***

| **Classifier** | **Accuracy** | **Sensitivity** | **Specificity** | **Precision** | **F1-score** | **MCC** | **Kappa** |
| --- | --- | --- | --- | --- | --- | --- | --- |
| **ECOC-SVM** | 0.7960 | 0.8244 | 0.9464 | 0.8184 | 0.8211 | 0.7676 | 0.3624 |
| **NB** | 0.6831 | 0.6933 | 0.9165 | 0.7085 | 0.6972 | 0.6170 | 0.0097 |
| **k-NN** | 0.9163 | 0.9124 | 0.9776 | 0.9320 | 0.9208 | 0.8997 | 0.7383 |
| **RF** | 0.9872 | 0.9891 | 0.9966 | 0.9892 | 0.9891 | 0.9857 | 0.9601 |
| **AB** | 0.9461 | 0.9521 | 0.9856 | 0.9548 | 0.9534 | 0.9341 | 0.8314 |
| **DNN** | 0.9344 | 0.9488 | 0.9828 | 0.9457 | 0.9448 | 0.9295 | 0.7948 |

***DatasetA + patch images***

1. ***Texture parameters***

| **Classifier** | **Accuracy** | **Sensitivity** | **Specificity** | **Precision** | **F1-score** | **MCC** | **Kappa** |
| --- | --- | --- | --- | --- | --- | --- | --- |
| **ECOC-SVM** | 0.6366 | 0.7038 | 0.9034 | 0.6819 | 0.6810 | 0.5959 | 0.1195 |
| **NB** | 0.4748 | 0.5722 | 0.8668 | 0.4945 | 0.5081 | 0.3980 | 0.3907 |
| **k-NN** | 0.8555 | 0.868 | 0.9613 | 0.8755 | 0.8709 | 0.8328 | 0.547 |
| **RF** | 0.7647 | 0.7792 | 0.9371 | 0.7922 | 0.7840 | 0.7228 | 0.2647 |
| **AB** | 0.7311 | 0.7381 | 0.9277 | 0.7723 | 0.7503 | 0.6825 | 0.1597 |
| **DNN** | 0.8866 | 0.9119 | 0.9694 | 0.9096 | 0.9085 | 0.88 | 0.6455 |

1. ***Color parameters***

| **Classifier** | **Accuracy** | **Sensitivity** | **Specificity** | **Precision** | **F1-score** | **MCC** | **Kappa** |
| --- | --- | --- | --- | --- | --- | --- | --- |
| **ECOC-SVM** | 0.5819 | 0.6248 | 0.8887 | 0.6277 | 0.6258 | 0.5149 | 0.2346 |
| **NB** | 0.4202 | 0.4716 | 0.8517 | 0.4205 | 0.4267 | 0.2939 | 0.4481 |
| **k-NN** | 0.6029 | 0.6253 | 0.8948 | 0.6266 | 0.6222 | 0.52 | 0.1941 |
| **RF** | 0.6303 | 0.651 | 0.9006 | 0.6859 | 0.6658 | 0.5686 | 0.1345 |
| **AB** | 0.5987 | 0.6282 | 0.8926 | 0.6522 | 0.6384 | 0.5324 | 0.2025 |
| **DNN** | 0.9559 | 0.9646 | 0.9878 | 0.9673 | 0.9658 | 0.9539 | 0.8621 |

1. ***Spectrum parameters***

| **Classifier** | **Accuracy** | **Sensitivity** | **Specificity** | **Precision** | **F1-score** | **MCC** | **Kappa** |
| --- | --- | --- | --- | --- | --- | --- | --- |
| **ECOC-SVM** | 0.5273 | 0.5245 | 0.8737 | 0.5547 | 0.52228 | 0.4095 | 0.323 |
| **NB** | 0.4496 | 0.4897 | 0.8575 | 0.4583 | 0.4711 | 0.3307 | 0.4186 |
| **k-NN** | 0.6744 | 0.6974 | 0.9137 | 0.7010 | 0.6981 | 0.613 | 0.0173 |
| **RF** | 0.7080 | 0.7092 | 0.9205 | 0.7729 | 0.7153 | 0.654 | 0.0874 |
| **AB** | 0.6576 | 0.6595 | 0.9073 | 0.7057 | 0.6699 | 0.5873 | 0.0655 |
| **DNN** | 0.7206 | 0.7469 | 0.9278 | 0.7593 | 0.7392 | 0.676 | 0.1268 |

1. ***Combining of texture and color parameters***

| **Classifier** | **Accuracy** | **Sensitivity** | **Specificity** | **Precision** | **F1-score** | **MCC** | **Kappa** |
| --- | --- | --- | --- | --- | --- | --- | --- |
| **ECOC-SVM** | 0.6702 | 0.7004 | 0.9116 | 0.719 | 0.7085 | 0.6212 | 0.0298 |
| **NB** | 0.5504 | 0.6419 | 0.8863 | 0.5995 | 0.5893 | 0.5028 | 0.2882 |
| **k-NN** | 0.8613 | 0.8684 | 0.9626 | 0.8832 | 0.8743 | 0.8384 | 0.5667 |
| **RF** | 0.7752 | 0.7936 | 0.94 | 0.8003 | 0.7964 | 0.737 | 0.2975 |
| **AB** | 0.7521 | 0.7772 | 0.9333 | 0.791 | 0.7837 | 0.7175 | 0.2253 |
| **DNN** | 0.9265 | 0.921 | 0.9794 | 0.9527 | 0.932 | 0.9158 | 0.7702 |

1. ***Combining of texture and spectrum parameters***

| **Classifier** | **Accuracy** | **Sensitivity** | **Specificity** | **Precision** | **F1-score** | **MCC** | **Kappa** |
| --- | --- | --- | --- | --- | --- | --- | --- |
| **ECOC-SVM** | 0.5336 | 0.5357 | 0.8739 | 0.6039 | 0.5483 | 0.4381 | 0.3139 |
| **NB** | 0.4643 | 0.4787 | 0.8588 | 0.484 | 0.4796 | 0.3405 | 0.4027 |
| **k-NN** | 0.7794 | 0.7920 | 0.9414 | 0.8054 | 0.7976 | 0.74 | 0.3107 |
| **RF** | 0.8046 | 0.8141 | 0.9471 | 0.8377 | 0.8234 | 0.773 | 0.3894 |
| **AB** | 0.7542 | 0.7618 | 0.9335 | 0.7987 | 0.7763 | 0.7132 | 0.2319 |
| **DNN** | 0.666 | 0.6069 | 0.9087 | 0.7517 | 0.6401 | 0.5775 | 0.042 |

1. ***Combining of color and spectrum parameters***

| **Classifier** | **Accuracy** | **Sensitivity** | **Specificity** | **Precision** | **F1-score** | **MCC** | **Kappa** |
| --- | --- | --- | --- | --- | --- | --- | --- |
| **ECOC-SVM** | 0.521 | 0.5256 | 0.8715 | 0.5515 | 0.5244 | 0.4071 | 0.3319 |
| **NB** | 0.2962 | 0.2075 | 0.8029 | nan | nan | nan | 0.5453 |
| **k-NN** | 0.7857 | 0.82 | 0.9425 | 0.8155 | 0.8170 | 0.7602 | 0.3304 |
| **RF** | 0.7668 | 0.7833 | 0.9374 | 0.7961 | 0.7858 | 0.7263 | 0.2713 |
| **AB** | 0.7185 | 0.7487 | 0.9255 | 0.7464 | 0.7472 | 0.6729 | 0.1203 |
| **DNN** | 0.6387 | 0.5433 | 0.8994 | nan | nan | nan | 0.1144 |

1. ***Full parameters***

| **Classifier** | **Accuracy** | **Sensitivity** | **Specificity** | **Precision** | **F1-score** | **MCC** | **Kappa** |
| --- | --- | --- | --- | --- | --- | --- | --- |
| **ECOC-SVM** | 0.5798 | 0.5945 | 0.8877 | 0.6231 | 0.6032 | 0.4960 | 0.2384 |
| **NB** | 0.2857 | 0.2 | 0.8 | Nan | Nan | Nan | 0.552 |
| **k-NN** | 0.792 | 0.8126 | 0.9439 | 0.8262 | 0.8188 | 0.7634 | 0.3501 |
| **RF** | 0.8109 | 0.8281 | 0.9485 | 0.8508 | 0.8374 | 0.7878 | 0.4091 |
| **AB** | 0.7857 | 0.8015 | 0.9424 | 0.8188 | 0.8091 | 0.7527 | 0.3304 |
| **DNN** | 0.7521 | 0.7424 | 0.9336 | 0.826 | 0.7654 | 0.715 | 0.2253 |

***DatasetB + patch images***

1. ***Texture parameters***

| **Classifier** | **Accuracy** | **Sensitivity** | **Specificity** | **Precision** | **F1-score** | **MCC** | **Kappa** |
| --- | --- | --- | --- | --- | --- | --- | --- |
| **ECOC-SVM** | 0.7331 | 0.7639 | 0.9299 | 0.7555 | 0.7593 | 0.6893 | 0.166 |
| **NB** | 0.8546 | 0.86 | 0.9613 | 0.8766 | 0.8669 | 0.8297 | 0.5455 |
| **k-NN** | 0.9412 | 0.9311 | 0.9843 | 0.9512 | 0.9397 | 0.9254 | 0.8162 |
| **RF** | 0.928 | 0.9323 | 0.981 | 0.9355 | 0.9336 | 0.9149 | 0.7749 |
| **AB** | 0.7686 | 0.793 | 0.9386 | 0.8043 | 0.7968 | 0.7372 | 0.2769 |
| **DNN** | 0.9095 | 0.9111 | 0.9758 | 0.9267 | 0.9163 | 0.8947 | 0.7173 |

1. ***Color parameters***

| **Classifier** | **Accuracy** | **Sensitivity** | **Specificity** | **Precision** | **F1-score** | **MCC** | **Kappa** |
| --- | --- | --- | --- | --- | --- | --- | --- |
| **ECOC-SVM** | 0.5449 | 0.5902 | 0.8822 | 0.5661 | 0.5696 | 0.4592 | 0.2969 |
| **NB** | 0.8486 | 0.8601 | 0.9594 | 0.8839 | 0.8682 | 0.8313 | 0.527 |
| **k-NN** | 0.9708 | 0.9707 | 0.9923 | 0.9727 | 0.9717 | 0.9641 | 0.9087 |
| **RF** | 0.9767 | 0.9783 | 0.9938 | 0.9796 | 0.9789 | 0.9728 | 0.9271 |
| **AB** | 0.9071 | 0.9204 | 0.9751 | 0.9231 | 0.9215 | 0.8969 | 0.7097 |
| **DNN** | 0.9273 | 0.9392 | 0.9802 | 0.946 | 0.9418 | 0.9228 | 0.7727 |

1. ***Spectrum parameters***

| **Classifier** | **Accuracy** | **Sensitivity** | **Specificity** | **Precision** | **F1-score** | **MCC** | **Kappa** |
| --- | --- | --- | --- | --- | --- | --- | --- |
| **ECOC-SVM** | 0.4958 | 0.5049 | 0.8660 | 0.5450 | 0.5077 | 0.3866 | 0.3653 |
| **NB** | 0.4819 | 0.5279 | 0.8665 | 0.5088 | 0.5033 | 0.3834 | 0.3824 |
| **k-NN** | 0.7982 | 0.8105 | 0.9472 | 0.8119 | 0.8105 | 0.7583 | 0.3693 |
| **RF** | 0.8159 | 0.8195 | 0.9508 | 0.8489 | 0.8278 | 0.7837 | 0.4248 |
| **AB** | 0.6193 | 0.6253 | 0.8973 | 0.7009 | 0.6327 | 0.5509 | 0.1593 |
| **DNN** | 0.5376 | 0.4972 | 0.8747 | 0.7533 | 0.5263 | 0.4771 | 0.3080 |

1. ***Combining of texture and color parameters***

| **Classifier** | **Accuracy** | **Sensitivity** | **Specificity** | **Precision** | **F1-score** | **MCC** | **Kappa** |
| --- | --- | --- | --- | --- | --- | --- | --- |
| **ECOC-SVM** | 0.7516 | 0.7793 | 0.9346 | 0.7753 | 0.7758 | 0.7117 | 0.2236 |
| **NB** | 0.8521 | 0.8643 | 0.9603 | 8845 | 0.8719 | 0.8346 | 0.5379 |
| **k-NN** | 0.9224 | 0.9163 | 0.9792 | 0.9382 | 0.9256 | 0.9063 | 0.7575 |
| **RF** | 0.9805 | 0.9836 | 0.9948 | 0.9835 | 0.9835 | 0.9784 | 0.9391 |
| **AB** | 0.9228 | 0.9325 | 0.9792 | 0.938 | 0.935 | 0.9146 | 0.7586 |
| **DNN** | 0.9861 | 0.9884 | 0.9962 | 0.9896 | 0.9890 | 0.9853 | 0.9565 |

1. ***Combining of texture and spectrum parameters***

| **Classifier** | **Accuracy** | **Sensitivity** | **Specificity** | **Precision** | **F1-score** | **MCC** | **Kappa** |
| --- | --- | --- | --- | --- | --- | --- | --- |
| **ECOC-SVM** | 0.532 | 0.5673 | 0.8768 | 0.5659 | 0.5640 | 0.4429 | 0.3162 |
| **NB** | 0.5915 | 0.625 | 0.8928 | 0.6211 | 0.6171 | 0.5152 | 0.2166 |
| **k-NN** | 0.8838 | 0.8801 | 0.969 | 0.9035 | 0.8902 | 0.8607 | 0.6368 |
| **RF** | 0.9235 | 0.9306 | 0.9798 | 0.9328 | 0.9316 | 0.9115 | 0.7608 |
| **AB** | 0.7624 | 0.7862 | 0.9367 | 0.8011 | 0.7916 | 0.7303 | 0.2574 |
| **DNN** | 0.5623 | 0.5373 | 0.8828 | 0.7007 | 0.5644 | 0.4841 | 0.2689 |

1. ***Combining of color and spectrum parameters***

| **Classifier** | **Accuracy** | **Sensitivity** | **Specificity** | **Precision** | **F1-score** | **MCC** | **Kappa** |
| --- | --- | --- | --- | --- | --- | --- | --- |
| **ECOC-SVM** | 0.5334 | 0.5452 | 0.8763 | 0.5813 | 0.5515 | 0.4366 | 0.3142 |
| **NB** | 0.5219 | 0.5347 | 0.8771 | 0.5323 | 0.5325 | 0.4102 | 0.3307 |
| **k-NN** | 0.9043 | 0.9163 | 0.9746 | 0.978 | 0.9169 | 0.8917 | 0.7010 |
| **RF** | 0.9656 | 0.97 | 0.9909 | 0.9698 | 0.9699 | 0.9608 | 0.8924 |
| **AB** | 0.8817 | 0.8915 | 0.9683 | 0.9017 | 0.8981 | 0.8668 | 0.6303 |
| **DNN** | 0.5971 | 0.5526 | 0.8893 | 0.8294 | 0.6022 | 0.5616 | 0.2058 |

1. ***Full parameters***

| **Classifier** | **Accuracy** | **Sensitivity** | **Specificity** | **Precision** | **F1-score** | **MCC** | **Kappa** |
| --- | --- | --- | --- | --- | --- | --- | --- |
| **ECOC-SVM** | 0.7258 | 0.7677 | 0.9278 | 0.758 | 0.7618 | 0.6904 | 0.1432 |
| **NB** | 0.667 | 0.6821 | 0.9126 | 0.681 | 0.6779 | 0.5936 | 0.039 |
| **k-NN** | 0.9294 | 0.9217 | 0.9809 | 0.9455 | 0.9319 | 0.9144 | 0.7793 |
| **RF** | 0.9739 | 0.9776 | 0.993 | 0.9786 | 0.9781 | 0.9711 | 0.9184 |
| **AB** | 0.9193 | 0.9277 | 0.9784 | 0.9331 | 0.9302 | 0.9089 | 0.7477 |
| **DNN** | 0.9694 | 0.9759 | 0.9921 | 0.9716 | 0.9735 | 0.9656 | 0.9043 |
